# Evaluating adverse events in databases

**DOI:** 10.1002/prp2.1129

**Published:** 2023-08-17

**Authors:** Katie Lovell, Steven R. Feldman

**Affiliations:** ^1^ Department of Dermatology, Center for Dermatology Research Wake Forest University School of Medicine Winston‐Salem North Carolina USA; ^2^ Department of Dermatology Wake Forest University School of Medicine Winston‐Salem North Carolina USA; ^3^ Department of Pathology Wake Forest University School of Medicine Winston‐Salem North Carolina USA; ^4^ Department of Social Sciences & Health Policy Wake Forest University School of Medicine Winston‐Salem North Carolina USA

**Keywords:** adverse events, drug safety, IL‐23 inhibitors, spontaneous reporting database

**Abbreviations**
IL‐23: interleukin‐23

In this issue, Calapai et al. reported a potential association between oncologic adverse reactions and interleukin‐23 (IL‐23)^1^ inhibitor use in psoriasis patients using the EudraVigilance database for spontaneous adverse event reports.^2^ The study evaluated suspected adverse events from 2020 to 2022 with a reported 53 events related to oncologic disease development following the use of IL‐23 blockers: guselkumab, risankizumab, or tildrakizumab.^2^ The reporting odds ratio (ROR) for risankizumab and tildrakizumab was >1 when comparing each drug to the other two, concluding a potential association between IL‐23 inhibitors and the occurrence of oncologic adverse events.^2^ While adverse event reporting databases can be valuable resources to monitor drug safety, there are major limitations to drawing definite conclusions.

To determine with great confidence that a drug causes an adverse reaction, a randomized, placebo‐controlled trial can be done. Such trials would account for the potential random variation with which events occur. Unfortunately, randomized trials may be impractical due to the time and resources required, particularly for the detection of rare effects.^3^ An alternative design allowing for increased practicality while compromising on causality is the use of a prospective registry. A prospective registry that follows a substantial number of individuals, with a control and treatment group, can provide valuable insights even without randomization. While the lack of randomization may result in the introduction of some bias, the incorporation of a control group allows for meaningful comparisons between events related to the natural course of disease versus adverse drug events.

The use of claims data is another potential method to evaluate for an association between a drug and adverse effects in real‐world health practices. Claims data allow for a reasonable estimate of how many individuals are exposed to a drug of interest versus those unexposed and how many develop the outcome of interest. Assessments based on claims data, like prospective cohort studies, suffer from the potential for bias in who is treated; if patients at high risk for a particular event are more likely treated with a particular medication (because that medication does not increase the risk of that event), a high event rate may become associated with the treatment. Another limitation of claims data is that only events requiring medical attention are reported in claims databases; this may not be a critical problem, as we may be most concerned with major adverse events that would result in medical care.

The approach adopted by Calapai et al. was the use of a spontaneous reporting database. While these databases are important for pharmacovigilance,^3^ severe limitations exist for drawing definitive conclusions. One limitation is the lack of controls in this type of study, which makes it difficult to distinguish adverse events related to the drug versus the natural course of events in a population. An increased risk between psoriasis and subsequent overall malignancy has been identified in many prior studies,^4^, ^5^, ^6^, ^7^ with persistent immune activation and inflammatory response as possible mechanisms. Thus, the utilization of a control group in this population is needed to strengthen the claim that increased oncologic events are due to a biologic medication rather than a natural progression of the disease. Systematic biases may confound the results; for example, drugs that block IL‐23 have no contraindication for malignancy and may be first line choices for patients at high risk of new or recurrent malignancy; identifying a high rate of malignancy with these agents may have far more to do with the treated population than with an effect of the drug.

The use of a spontaneous reporting database results in a lack of a denominator for calculating an incidence rate; therefore, risk cannot be determined without knowledge of the actual number of individuals exposed. Additionally, several types of bias can result from the use of a spontaneous database study. The Weber and notoriety effects are two notable forms of selection bias often observed, and critically, only a fraction of the actual number of adverse events are reported.^3^ This can be secondary to selection bias or due to the lack of time for or knowledge of reporting processes among healthcare providers and the public. Thus, when relying on spontaneous reporting, we lack information on both the numerator and the denominator of risk for both the exposed and unexposed populations.

In general, IL‐23 inhibitors are safe drugs typically well tolerated by patients. While spontaneous reporting databases hold an important role in drug surveillance, it is important to understand the limitations of such studies in concluding the causation of adverse events. Clinical trials and prospective registry studies are currently not supportive of an increased risk of malignancy with IL‐23 blockade.^8^, ^9^

## AUTHOR CONTRIBUTIONS

Katie Lovell completed the preliminary research and wrote the manuscript. Steven R Feldman conceived the commentary, provided technical and grammatical feedback, and assisted in writing the manuscript.

## CONFLICT OF INTEREST STATEMENT

Feldman has received research, speaking, and/or consulting support from Eli Lilly and Company, GlaxoSmithKline/Stiefel, AbbVie, Janssen, Alovtech, vTv Therapeutics, Bristol‐Myers Squibb, Samsung, Pfizer, Boehringer Ingelheim, Amgen, Dermavant, Arcutis, Novartis, Novan, UCB, Helsinn, Sun Pharma, Almirall, Galderma, Leo Pharma, Mylan, Celgene, Ortho Dermatology, Menlo, Merck & Co, Qurient, Forte, Arena, Biocon, Accordant, Argenx, Sanofi, Regeneron, the National Biological Corporation, Caremark, Teladoc, BMS, Ono, Micreos, Eurofins, Informa, UpToDate and the National Psoriasis Foundation. He is founder and part owner of Causa Research and holds stock in Sensal Health. Lovell has no conflicts of interest to disclose.

## ETHICS STATEMENT

This material is the authors' own original work, which has not been previously published elsewhere.

## NOMENCLATURE OF TARGETS AND LIGANDS

Key protein targets and ligands in this article are hyperlinked to corresponding entriesin http://www.guidetopharmacology.org, the common portal for data from the IUPHAR/BPS Guide to PHARMACOLOGY (Harding et al., 2018), and are permanently archived in the Concise Guide to PHARMACOLOGY 2019/20 (Alexander et al., 2019).

## Data Availability

Data sharing is not applicable to this article as no new data were created or analyzed in this study.
